# Biosynthesis of cellulose from *Ulva lactuca*, manufacture of nanocellulose and its application as antimicrobial polymer

**DOI:** 10.1038/s41598-023-37287-7

**Published:** 2023-06-22

**Authors:** Mostafa M. El-Sheekh, Wesam E. Yousuf, El-Refaie Kenawy, Tarek M. Mohamed

**Affiliations:** 1 Botany Department, Faculty of Science, Tanta University, Tanta, 31527 Egypt; 2 Biochemistry Division, Chemistry Department, Faculty of Science, Tanta University, Tanta, 31527 Egypt; 3 Polymer Research Group Chemistry Department, Faculty of Science, Tanta University, Tanta, 31527 Egypt

**Keywords:** Biochemistry, Microbiology

## Abstract

Green nanotechnology has recently been recognized as a more proper and safer tool for medical applications thanks to its natural reductions with low toxicity and avoidance of injurious chemicals. The macroalgal biomass was used for nanocellulose biosynthesis. Algae are abundant in the environment and have a high content of cellulose. In our study, we extracted parent cellulose from *Ulva lactuca* where consecutive treatments extracted cellulose to obtain an insoluble fraction rich in cellulose. The extracted cellulose has the same results obtained by matching it with reference cellulose, especially the same Fourier transform infrared (FTIR) and X-Ray diffraction (XRD) analysis peaks. Nanocellulose was synthesized from extracted cellulose with hydrolysis by sulfuric acid. Nanocellulose was examined by Scanning electron microscope (SEM) shown by a slab-like region as Fig. 4a and Energy dispersive X-ray (EDX) to examine the chemical composition. The size of nanocellulose in the range of 50 nm is calculated by XRD analysis. Antibacterial examination of nanocellulose was tested against Gram+ bacteria like *Staphylococcus aureus* (ATCC6538), *Klebsiella pneumonia* (ST627), and Gram-negative bacteria such as *Escherichia coli* (ATCC25922), and *coagulase-negative Staphylococci (CoNS)* to give 4.06, 4.66, 4.93 and 4.43 cm as respectively. Comparing the antibacterial effect of nanocellulose with some antibiotics and estimating minimal Inhibitory Concentration (MIC) of nanocellulose. We tested the influence of cellulose and nanocellulose on some fungi such as *Aspergillus flavus, Candida albicans,* and *Candida tropicalis*. These results demonstrate that nanocellulose could be developed as an excellent solution to these challenges, making nanocellulose extracted from natural algae a very important medical material that is compatible with sustainable development.

## Introduction

Antibacterial materials based on nanocellulose have a remarkable potential effect to be used as a wide range of components used in protective equipment (mainly in the healthcare sector), textiles, food packaging, paints, pharmaceutical movers, ultrafiltration, adsorbents, and wound plasters. The outstanding qualities of cellulose, such as renewability and environmental benefits, have led to its use in various industrial applications. On Earth, cellulose is the most abundant natural polysaccharide^1,2^. Being more adaptable than other biopolymers, cellulose itself is used in particular because it can be used for a variety of biomedical applications^3^. Nanocellulose seems to be a great choice as paternal materials because of its wide availability, cheapness, biocompatibility, low density, elasticity, nontoxicity, biodegradability, low thermal stability, and mechanical properties^4^. At this time, nanocellulose can be obtained from wood, algae, tunicates, and agro-industrial residue or biomass^5^. Cellulose's reactive properties stem from the presence of practical hydroxyl groups. In this way, cellulose can be chemically enhanced for use in other contexts^6^. Cellulose is a vital bio-based polymer that may be recycled into numerous products, including plastics, photographic films, and textiles^7^. Marine algae can produce greater quantities of deposited carbohydrates and are experts at growing in environments with limited growth supplies. They can do this by making efficient use of carbon dioxide, light, and inorganic nutrients, all of which are free to offer^8^. Cellulose is a major constituent of the cell walls of the seaweed *Ulva lactuca*, coupled by ulvan polymer^9^. The physical and mechanical properties of cellulose and its derivatives, such as biocompatible polymers, make them very sensitive to uses in the biomedical sector^10^. Using cellulose extracted from green algae and converting it into nanocellulose to treat some types of bacteria that cause harm to humans is one of the challenges that make humans able to use green chemistry. In recent years, cellulose derived from macroalgae has received more attention than it previously has due to its many proposed advantages over cellulose derived from land-based biomass, which is more suited for biological applications^11^. Bacteria are frequently growing and developing an increased struggle with antibiotics and decontaminators^12^. Fungal infections originating from *Candida albicans* are the main health difficulty. Subsequently, this microorganism can potentially cause life-threatening localized and systemic infections^13^. Rapid multiplication and drug resistance make some bacteria more contagious than viruses. As a result, there has to be constant development and exploration of new antimicrobial products^14^. Infections caused by *Staphylococcus aureus* are common and include acne, ulcers, impetigo, and a wide variety of pus-filled abscesses on wounds^15^, urinary tract infections are frequently initiated by *Klebsiella pneumonia*^16^, gastric infections are commonly caused by *Escherichia coli*^17^. Thus, it was essential to search for ecological solutions from the environment to challenge this tricky. nanocellulose can be an abundant applicant, for example, an antimicrobial sensitivity on microbes^18^. nanocellulose has been used as a transporter for diverse antibacterial causes, principal to the advance of antibacterial materials for several applications^19^. Nanocellulose has also been used for the production of antimicrobial applications^20^, hygienic materials^21^, larvicidal agents^22^, or wound healing^23,24^, depending on the sources and production method. Therefore, this research aims to extract cellulose from *Ulva lactuca* and characterize it, synthesize nanocellulose (NC) from cellulose extracted, then characterize it and test the antibacterial effect of nanocellulose on Gram (+ve) bacteria such as *Staphylococcus aureus* (ATCC6538) and *Klebsiella pneumonia* (*ST627*)*,* Gram (−ve) bacteria such as *Escherichia coli* (ATCC25922) and *coagulase-negative staphylococci (CoNS)* and some fungi such as *Aspergillus flavus, Candida albicans,* and *Candida tropicalis* were the essential appliance of collaboration among the nanocellulose and microbes is also clarified.

## Materials and methods

### Materials

Hydrogen peroxide (30%), hydrochloric acid (37%), sodium acetate (99%), sodium hydroxide (99%), Ethanol (99%), sulfuric acid (99%), sodium bicarbonate, antibiotic discs, Sabouraud Dextrose Agar and Agar media were purchased from El-Gomhouria chemicals Company, Egypt. And reference cellulose from Acros Organics Company, Germany.

### Collection of *Ulva lactuca*

*Ulva lactuca* was gathered in spring from the Abo Qir shore at Alexandria, Egypt. Once the *Ulva lactuca* had been collected, it was wiped multiple times with sea water to eliminate populations and epiphytes, finally washed with distilled water until it became clean, left to dry in the open air, and the samples were air dried, and then milled for 5 min at a size of roughly 0.5 mm using (Fritsch, Pulverisette 2, and Filtra vibracion S.L.). This produced a fine and homogeneous powder. For later analysis; we kept the milled seaweed samples at room temperature (25 °C) in sealed bags^25^.

### The separation of cellulose

The cellulose was harvested using a method similar to^26^ which is described as follows, albeit with a few tweaks to eliminate the necessity for sodium hypochlorite and methanol. The separation process is monitored as follows: *Ulva lactuca* was lyophilized (50 g), crushed to a fine powder using a hand mixer, and extracted using Soxhlet in ethanol (85%) for 24 h at 120 °C to exclude coloring and any oils. After discarding the liquid stage, the solid part was cleaned many times with ethanol (99%), then left to dry on a 37 °C stove for over 16 h. This sample was dried and then treated by being suspended in 400 mL of 4% hydrogen peroxide for 16 h at 80 °C. During that time, any leftover green pigments and other colored impurities were eliminated. After letting the mixture settle to ambient temperature, the residue was vortexed at 5000 rpm for approximately fifteen minutes before being discarded. Before being floated in 400 mL of 0.5 M sodium hydroxide, the material was washed with water till the pH of the effluents approached 7. The mixture was kept in an oven at 60 °C for 16 h. We centrifuged the mixture at 5000 rpm for 15 min after letting it settle to ambient temperature and then discarded the supernatant. The substance was subjected to repeated soakings until its pH exceeded 7, the same as that of the rinse water. Subsequently, 200 mL of 5% hydrochloric acid was added to the insoluble portion. After the mixture reached the boiling stage, it was cooled to 30 °C and kept at that temperature for 16 h. The supernatant was discarded following centrifugation, and the solution was spun at 5000 rpm for fifteen min. The solid component containing cellulose was obtained, frozen in dry circumstances to remove moisture, and then used after undergoing numerous cleanses in the water until the pH of the washing water approached 7.

### Chemical composition of extracted cellulose and *Ulva lactuca*

For the determination of the chemical build of *Ulva lactuca* before and after the steps of extraction of cellulose, different typical methods of the Technical Association of the Pulp and Paper Industry (TAPPI) were used^27^. Generally followed to estimate the content of cellulose, it has to be understood here as the carbohydrate fraction which is resistant to consecutive treatments.

### Synthesis of nanocellulose

The synthesis of nanocellulose by cellulose extracted from algae was performed as follows: cellulose (flip 5.00 g/50 mL of distilled water), and add 54 mL of sulfuric acid (95%) by titration, and the mixture was slowly infused. For 20 min, we subjected the suspension to continual magnetic stirring while heating it to 44 °C. Incorporating more ice-cold distilled water halted the hydrolysis process^28^ and sonication at 750 W^29^.

### Characterization of cellulose and nanocellulose

#### Fourier-transform infrared spectroscope (FT-IR)

The 4000–500 cm^−1^ FT-IR spectra of dry cellulose and nanocellulose were monitored at room temperature using a Perkin-Elmer Spectrum 100 FTIR equipped with a triglycine monitor and an attenuated total reflectance crystal device (ATR Golden Gate) from Tanta University's Scientific Research Centre and Measurements (SRCM). The water and carbon dioxide levels in the atmosphere were modified. Planned as a mean of 50 shooters, the spectra obtained resulted from an average of 50 trials.

#### Quantification of the temperature stability

We employed thermo-gravimetric analysis (TGA) (Perkin Elmer TGA 4000 Thermogravimetric analyzer) to foretell cellulose and nanocellulose properties that could withstand high temperatures. The thermal stability of the cellulosic compound was established due to free OH which is present in the nanocellulose surface^30^. At a heating rate of 50 °C/min, alumina plates containing around 5 mg of freeze-dried sample were heated in an environment of nitrogen gas from 50 to 800 °C. Once the temperature reached 800 °C, N_2_ was removed and replaced with O_2_, and the heat was maintained for 15 min. The maximum allowed flow rate is 50 mL/min. Mass remaining after heating samples to 800 °C in N_2_ was used to calculate burn and residue content, and ash content was calculated from this mass. The procedure was performed at the Department of Chemistry, Faculty of Science, Tanta University.

#### Morphological examination

Morphological studies were carried out by Ultrahigh-resolution scan electron microscope (SEM) utilizing a Hitachi S4800 at Five kilovolts was employed to experimentally examine the morphology of cellulose isolated from *Ulva lactuca* and nanocellulose and transmission electron microscopy (TEM, Jeol-2100) was used for examined nanocellulose after sonication. After being dried overnight, following the attachment of nanocellulose to the sample stand with dual carbon scotch tape, a 7 nm Pt/Pd layer was blazed over the entire thing with a Cressington 208 HR in a nitrogen atmosphere and the energy dispersive X-ray spectrometer (EDX) was employed on the EDXA apex to examine the morphology and structure of nanocellulose. Our three investigations were conducted at the Egyptian Council of Research Centers and Institute.

#### X-ray diffraction (XRD)

Using monochrome CuKa rays (k = 0.1541 nm) in the band 2θ = 10°–90°, an Empyrean PAN-analytical XRD operates at 45 kV and 45 mA with a scan of 1.0 min^−1^ was used to examine the crystalline nature and cellulose nanocrystals of the cellulose extracted and nanocellulose at ambient temperature. To determine the level of crystallinity, Crl(%) was determined by plugging the values into the Segal equation^31^.

$$Crl(\%)=\frac{{\mathrm{I}}_{200}-{\mathrm{I}}_{am}}{{\mathrm{I}}_{200}} \times 100$$

where I_200_ is the intensity of the crystalline peak at 2θ = 22° in agreement with the (200), and I_am_ is the intensity of the local minimum of the curve at 2θ = 15° in agreement with amorphous quotas of the cellulose and The Debye–Scherrer strategy has been used to determine particle size^32^

$$D=\frac{ 0.9 \lambda }{ \beta cos\theta }$$

where (λ) is the wavelength of X-Ray (0.1541 nm), (β) is FWHM (full width at half maximum), (θ) is the diffraction angle, and (D) is particle diameter size which is performed at the Council of Research Centers and Institute, Egypt.

### In vitro susceptibility test

#### Test organisms

Reference strains of pathogenic microorganisms such as *Staphylococcus aureus* (ATCC6538), *Klebsiella pneumonia* (*ST627*)*, Escherichia coli (*ATCC25922), and *coagulase-negative staphylococci (CoNS)* and some fungi such as *Aspergillus flavus, Candida albicans,* and *Candida tropicalis* were obtained from Department of Microbiology, Faculty of Science, Tanta University.

#### Investigation of the experiment organisms

A loopful of each bacterial test organism was taken from the standard culture of these organisms and then lined on nutrient agar. The achieved bacterial culture was inoculated in sterile saline, and the bacterial suspension was attuned to around 10^5^ CFU/mL using MacFarland standards^33^. Mueller–Hinton agar media with 5 mm diameter wells were created using the well diffusion agar method, and 10^5^ CFU/mL of tested bacteria solution was plated using a sterile swab. The wells were then supplied with 1 micron of suspended cellulose and nanocellulose and 2 μg of antibacterial agent. After 24 h of incubation at 37 °C, the zone of growth inhibition was determined using a ruler. This technique was carried out thrice^34^. While *Aspergillus flavus, Candida albicans,* and *Candida tropicalis* were cultivated on Sabouraud Dextrose Agar (SDA) for 24 h at 37 °C before testing. The achieved inoculum was treated by suspending some colonies in at least 3 mL of sterile distilled water. After melting 20 mL of (SDA), cooling it to 55 °C, and inoculating it with 1 mL of the organism suspension, the turbidity of the mixture was adjusted with a spectrophotometer set to 530 nm to achieve a final concentration matching that of a 0.5 McFarland standard (0.5–2.5 × 10^3^). Once the agar with the inoculum had cooled, it was transferred to the assay plate. Agar was allowed to set, two wells were drilled into the plate, and 1 micron of cellulose and nanocellulose were added to each. Next, we insulated the plates at 35 degrees Celsius for 24 h. Each test should be taken thrice, and zones should be measured using a ruler^34^.

#### Comparison between the antibacterial effect of nanocellulose and antibiotics

For comparison between the antibacterial effect of nanocellulose and antibiotics, where the antibiotics applied in our study were chosen to cover approximately all the different antibiotic classes suitable for these microorganisms: *Staphylococcus aureus* (ATCC6538), *Klebsiella pneumonia* (ST627), *Escherichia coli* (ATCC25922) and *coagulase-negative staphylococci (CoNS)*. The antibiotics tested with their antimicrobial subclasses and the disc concentration is tabulated in Table 1. All samples were analyzed statistically, and significant differences were determined using a one-way ANOVA test. The significance of differences was determined for each group by comparing results obtained in the test samples with the result obtained for the corresponding nanocellulose sample as control (difference between the zone obtained with nanocellulose and the zone obtained with tested antibiotics on used microorganism), as well as the differences obtained between samples, for the various times of each of the three independent assays. Any p-value under 0.05 was recorded as statistically significant.

**Table 1:** The antibiotics used in their family classes along with the discs concentration against *Staphylococcus aureus* (ATCC6538), *Klebsiella pneumonia* (*ST627*)*, Escherichia coli* (ATCC25922), and *coagulase-negative staphylococci (CoNS).*

Family classes	Antibiotics
Macrolides	Azithromycin (AZM)
Lincosamides	Clindamycin (CD)
Aminoglycosides	Gentamicin (GEN)
Phenicols	Chloramphenicol (C)
4th generation cephalosporin	Cefepime (CMP)

### Nanocellulose minimum inhibitory concentration (MIC)

The typical serial dilution technique was used to study the antimicrobial efficacy of nanocellulose by evaluating the inhibition zone of microorganisms in the nutrient agar around the well. Serial dilutions of nanocellulose in concentrations ranging from 2 to 0.03 mg/mL with adjusted bacterial concentration (10^5^ CFU/mL) McFarland’s standard were used to determine the MIC of nanocellulose after inoculation of Petri dishes with tested bacteria. We distributed different dilutions of nanocellulose in each well, 0.5 μg/well. The nanocellulose is dissolved in sterile distilled water, and then each dish is incubated at 37 °C for 24 h. The MIC is the nanocellulose concentration at which there is no zone surrounding the well^35^, and this procedure is repeated 3 times.

## Result and discussion

**Figure 1:**
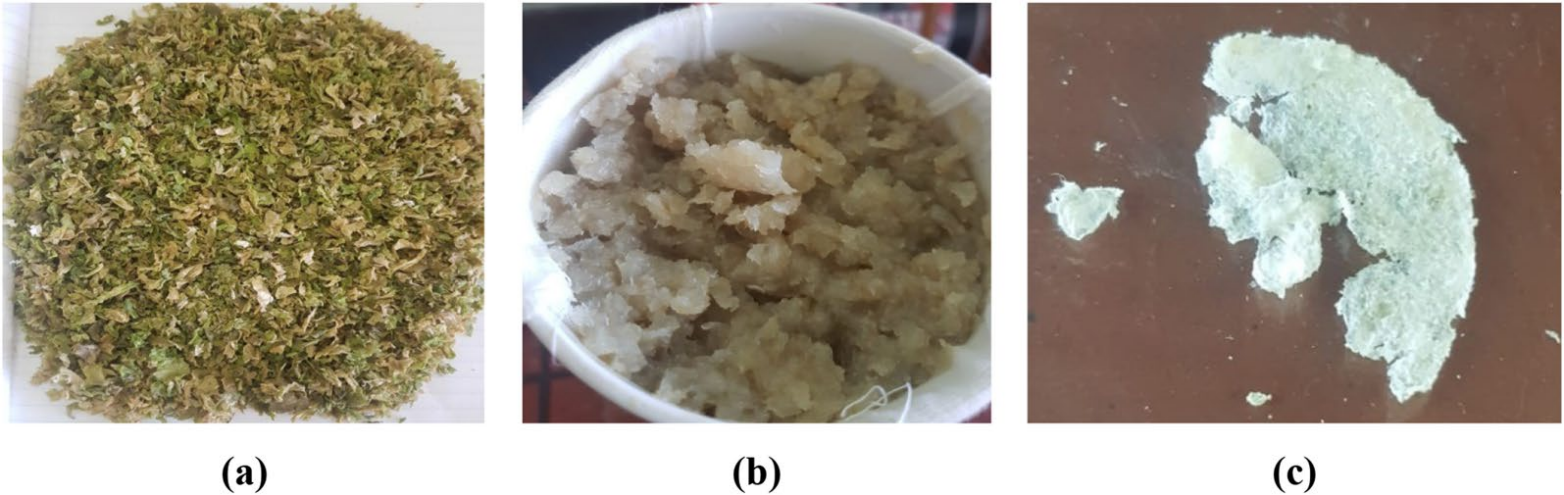
(**a**) *Ulva lactuca*, (**b**) cellulose as insoluble fraction, (**c**) cellulose dry weight.

*Ulva lactuca* green macroalgae were harvested to extract cellulose as shown in Fig. 1, creation and characterization of nanocellulose from harvested cellulose; assessment of the antibacterial vigor of extracted cellulose and nanocellulose against certain bacteria; these are the 3 main targets of this work. Initially, cellulose was extracted using multiple methodologies that included successive treatment of the biomass, resulting in the cellulose being separated as an insoluble fraction. Making nanocellulose from the isolated cellulose allowed the completion of the subsequent step.

### The separation of cellulose

Initial cellulose extraction yielded about 1.4 g from 50.0 g of total dry biomass for a harvest index of 2.8% based on the dry weight. This rate is comparable to that reported for the isolation of *Ulva lactuca* from French samples^36^. *Ulva lactuca's* chemical nature is influenced by its natural habitat, and cannot be directly matched to *Ulva lactuca* harvested in other locations. A further aspect that significantly affects the chemical nature of algae is the biological process of the individual algae^37^. Individuals of *Ulva lactuca* secondhand for this investigation were harvested in the spring when they had reached full maturation. Cellulose, hemicellulose, lignin, pectin, and wax are the primary components of the natural fibers that are extracted from algae. Cellulose can be found in both crystalline and amorphous phases, with the crystalline phase possessing a concentration distribution that is significantly higher than that of the amorphous phase. Within the structure of the fiber, cellulose is encased in a hemicellulose and lignin modeling of variable configuration, which varies depending on the type of fiber being examined. One might also refer to this as a natural composite^38^. Lignin is amorphous in its natural state and dissolves in hot alkali, but cellulose is resistant to the effects of strong alkali^39^. After being treated with sodium hydroxide at a concentration of 0.5 M, the microcellulose or cellulose microfibrils in the *Ulva lactuca* fibers remained securely connected with one another. The application of this step had the desired effect of partially separating the microfibrils from the fiber bundles, which indicated that the majority of the lignin was dissolved in the alkaline solution.

### Characterization of the extracted cellulose and nanocellulose

#### Chemical analysis of *Ulva lactuca* and extracted cellulose

By (TAPPI) we estimated the proportion of isolate cellulose from the obtained *Ulva lactuca* by removing lignin, hemicellulose, and other impurities as shown in Table 2, where *Ulva lactuca* dry powder consisted of 30.1% cellulose, 21.5% hemicellulose, 11.7% lignin, where cellulose, the main component of the cell wall, is more abundant in green algae^40^. After the steps of extraction of cellulose, the extractives such as pectin and wax were removed without significantly affecting the lignin, hemicellulose, and cellulose content of the fiber. Almost 80.7% of lignin was removed after all steps of treatment as shown in Table 2. The cellulose content was found to be 90.3%, and the content of lignin and hemicellulose was also partially reduced as a result of treatment. The percentage of ash in *Ulva lactuca* is higher than that found after the ashing of extracted cellulose. In general, macroalgae are characterized by high ash content and a very large percentage of mineral salts that is much higher than for vascular plants. When algae are collected, they contain sand and carbonated deposits. These impurities are largely eliminated during cleaning, but the residual contaminants contribute to the high percentage of ash.

**Table 2:** Chemical composition of *Ulva lactuca* and extracted cellulose.

	*Ulva lactuca*	Extracted cellulose
Cellulose	30.1 ± 1.6	90.2 ± 1.1
Hemicellulose	21.5 ± 1.2	8.2 ± 0.9
Lignin	11.7 ± 1.2	1.02 ± 0.1
Ash	19.21 ± 1.1	3.23 ± 0.07

#### FT-IR of the extracted cellulose and nanocellulose

**Figure 2:**
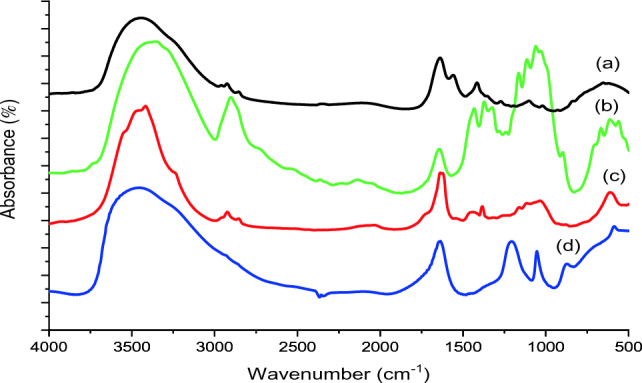
FTIR of (a) *Ulva lactuca*, (b) reference cellulose, (c) extracted cellulose, (d) nanocellulose.

The extracted cellulose and reference cellulose which it was selected from a known commercial source until it was compared with cellulose detached from *Ulva lactuca*, to ensure the purity of the extracted cellulose and the degree of elimination of other compounds present with cellulose in the raw material, and it can be matched with the reference cellulose that has known peaks, all were examined by FT-IR, which were measured at ambient temperature to be between 400 and 500 cm^−1^. The FT-IR spectra of the two types of cellulose reveal several characteristic crests aimed at polysaccharides and show matching in basic peaks, as shown in Fig. 2b,c.

The functional groups of the active components were analyzed by the spectrum of the FTIR Fig. 2 and compared to the measured values of the infrared absorption bands. The OH stretching of the aromatic rings, along with the presence of alcohol and phenol groups, led to the formation of the absorption peaks that occurred at 3448.94 cm^−1^^41^. The stretching vibration of the carbon-hydrogen bond in alkenes and ether is shown by a peak at 2925.02 cm^−1^^42^. There were bands at 1101.57 cm^−1^ coming from aliphatic amines and carboxylic acids, and amide I bands at 1638.65 cm^−1^ (C=O stretch of the ester group), amide II bands at 1559.76 cm^−1^ (N H bending)^43^, and the presences of peak around this peak refer to the presences of lignin in *Ulva lactuca*^44^. The bands of absorption at 653.70 cm^−1^ (Phaeophyta and Chlorophyta) showed stretching of C=S, indicating the presence of sulfides in the *Ulva lactuca*. Based on these findings, *Ulva lactuca* was discovered to contain a wide range of phytochemicals, including phenol, alcohol, lipids, proteins, and fatty acids compounds.

The hydrophilic properties of cellulosic materials are linked to the broad bands distinguished at 3500–3200 cm^−1^, and 1637 cm^−1^, which were recognized as the hydrogen bond of OH stretching and asymmetric elongating of the adsorbed water^45^ and O–H stretching vibration of cellulose molecules correspond to the groups between 3000 and 2800 cm^−1^^2^. H–C–H hairpin waves and H–C–H apex waves are associated with the crests at 1445 cm^−1^ and 1383 cm^−1^, respectively. The symmetric elongating of C–O–C connections accounts for the major peaks of about 1034–1113 cm^−1^^46^. Biologically significant amounts of polymeric polysaccharides, such as cellulose are indicated by the attendance of a modest peak at 895 cm^−1^, which is indicative of the beta-glycosidic bond^47^. Particularly noteworthy, the bands display no mark of the actuality of characteristic peaks between 1220 and 840–845 cm^−1^. Differentiating the polysaccharide ulvan, the vital part of *Ulva lactuca*, from these peaks relates to C–O–S squeezing and S=O squeezing^26^. Therefore, the disappearance of these peaks demonstrates that the targeted separation technique successfully removed ulvan from the cellulose proportion. The absence of a frequency at 1557 cm^−1^ indicative of N–H stretching amino acid also confirmed which cellulose specimens had no protein which appeared in 1559.76 cm^−1^ in *Ulva lactuca* chart as shown in Fig. 2a. The C=C extending in oxycellulose is responsible for the slight peak seen at 1636 cm^−1^^48^, demonstrating that cellulose oxidation yields oxycellulose occurred during the extraction, probably as a result of the hydrogen peroxide blenching phase. It's also likely that xyloglucan oxidation has developed. The 1113 cm^−1^ peak is consistent across all samples and can be attributed to the presence of glycosidic ether connections (C–O–C) between the anhydrous-glucopyranose ring skeleton and the glycosidic linkages between the anhydroglucose rings in the cellulose chains^45^. Both the cellulose and nanocellulose samples that were isolated showed all the characteristic peaks for cellulose with no pronounced changes.

#### Nanocellulose and isolated cellulose thermal evaluation

**Figure 3:**
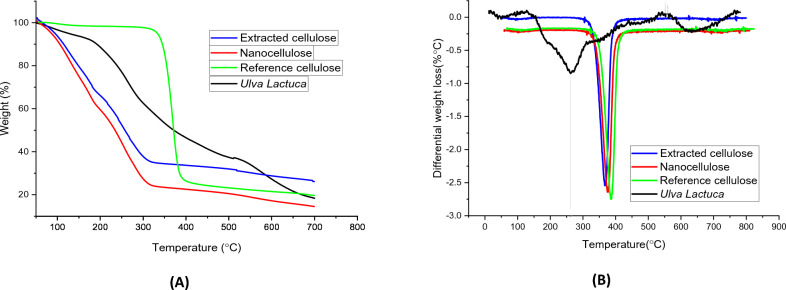
(**A**) TGA and (**B**) DTG curves showing the weight loss versus temperature.

TGA was used to assess the grilled *Ulva lactuca* that came before any processing, the cellulose that was taken from it, and the nanocellulose that has been designed from it, ash content is assumed in Table 2 and the TGA and DTG curves are exposed in Fig. 3A,B. At temperatures up to 100 °C, the extracted cellulose components lose bulk, and evaporation of water from residual nanocellulose or low-molecular-weight compounds after separation techniques are used accounts for the nearly imperceptible curve degradation within the temperature range of 25–120 °C^49^ for use in conditions where the temperature can reach 100 °C. This is typically brought on by the evaporation of adsorbed water. At temperatures above 320 °C, cellulose undergoes thermal exhaustion and undergoes the greatest loss of mass.

This reveals that *Ulva lactuca* cellulose is stable in terms of temperature up until this point. It was once believed that different species of seaweed have varying degrees of thermal stability for their cellulose^50^, and the average temperature falls between 250 and 300 °C. The deprivation occurs between 120 and 180 °C, although the larger pyrolysis process is evident in the curve. While thermal deterioration occurs over a broad temperature range, a lot of the time is spent in the hydrolysis phase. The substantially varied particle crystallinities between the samples are the primary cause of this difference. Under the influence of SO_4_^2−^ groups, we believe dehydration occurred at the cellulose chain units directly at a lower temperature; later, we believe a degradation reaction occurred at the polymer backbone, which was not in touch with the activated carbon, or at the interior of the cellulose crystal, possibly backed by the formation of some charred ash^51^. Based on these data, higher crystalline content and lower concentration of SO_4_^2−^ groups will improve the heat stability of nanocellulose. DTG curves of the extracted cellulose show a single peak that corresponds to cellulose breakdown. The peak values are *Ulva lactuca* 255 °C, extracted cellulose 360 °C, nanocellulose 375 °C and reference cellulose 380 °C.

#### Morphological examination

**Figure 4:**
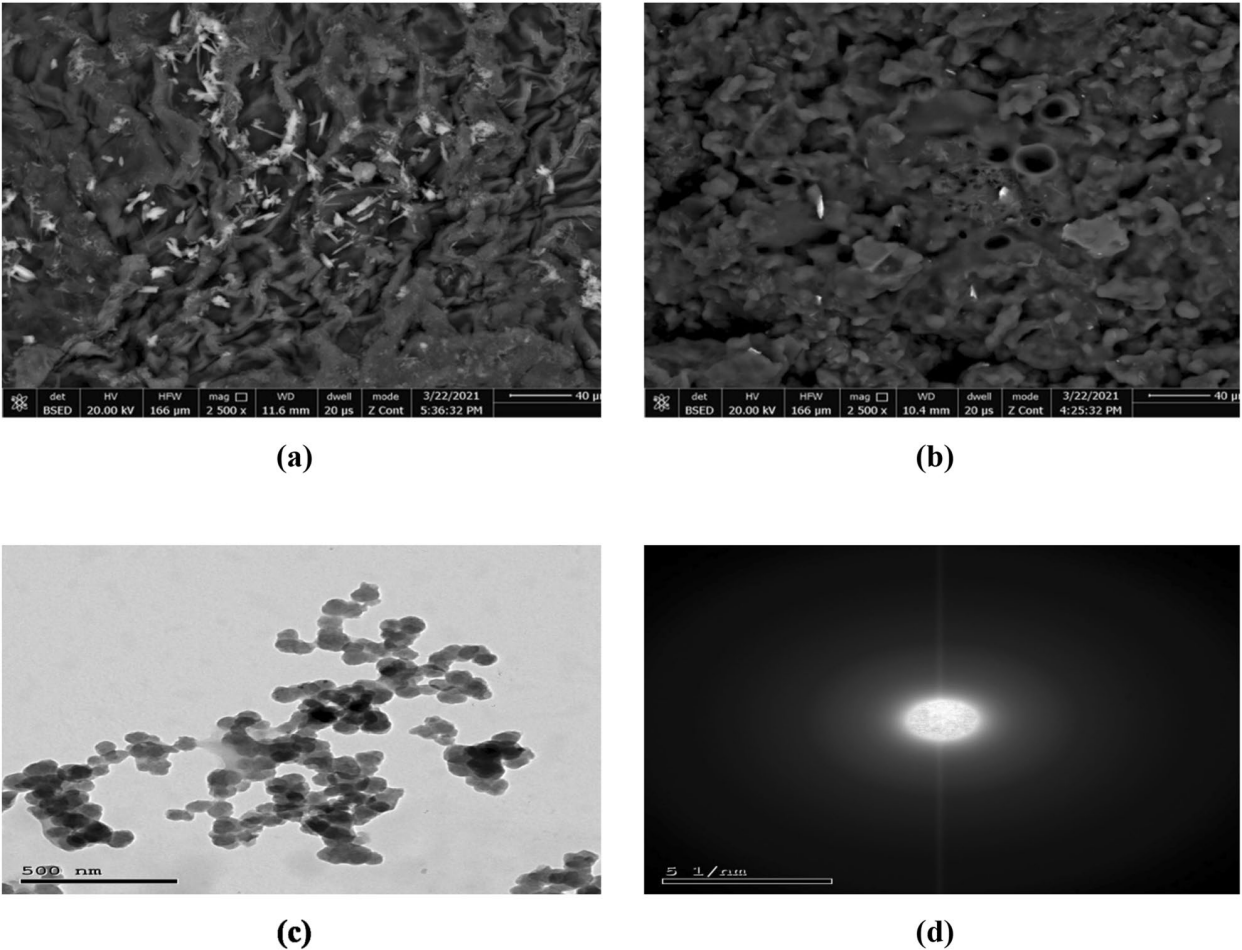
SEM (**a**) Extracted cellulose, (**b**) nanocellulose and (**c**) TEM images of spherical nanocellulose (**d**) electron diffraction pattern obtained from (**c**).

When the nanocellulose that was made from the extracted cellulose was examined under an SEM, it was found that the hydrolysis process had successfully separated the cellulose into individual fibers as Fig. 4a,b, Single fibrils with dimensions within the realm of nanometers (thickness besides length) and expanses with a slab-like organization, as seen by scanning electron microscopy. Nanofibers of nanocellulose were observed in a highly organized and smooth deagglomeration state, whereas cellulose and nanocellulose are displayed in a chaotic agglomeration position in SEM pictures^52^, as shown in Fig. 4b. The slab-like shape may result from cellulose not defibrillated during the homogenization manner^26^. Following alkalization under the conditions described in the prior section, sonication produced a spherical shape on the nanoscale with a diameter of less than 50 nm, and this proves that, nanocellulose made with sulfuric acid had smaller particles, increasing its surface area above samples made with other acids^53^, as observed in transmission electron microscopy (TEM) Fig. 4c, according to the XRD results given in Table 5, the average size of nanocllulose particles is 30.5024 nm. Taking TEM pictures of nanocellulose throughout the material allowed us to verify its spherical form Fig. 4c and show the electron diffraction pattern Fig. 4d obtained from the main image reveals a relatively medium diffraction ring due to the existence of residual amorphous cellulose. In other areas, however, it was clear that the cellulose nanosphere nanoparticles were dispersed throughout the amorphous matrix, a phenomenon known as residual amorphous cellulose.

Crystalline and amorphous phases of cellulose, found in natural fibers, are well recognized. The inherently hydrophilic nature of cellulose led to the agglomeration of cellulose nanospheres Fig. 4c^54^. Alternatively, the freeze-drying process done before the SEM investigation may have induced the slab-like patterns to reflect aggregated cellulose fibrils^55^. It is suspected that xyloglucan, a xylose-glucose polymer found in cellulose, is responsible for the slab-like sections^56^. Because they can pack tightly, linear polysaccharides like cellulose can form nanofibrils, but branching polysaccharides like xyloglucan often do not. Close packing of the molecules is necessary for the formation of these nanofibrils. The existence of both nanofibrils and slab-like regions, with the nano-fibers composed of cellulose, the slab-like formation made of xyloglucan, is another indicator that nanocellulose is a blend of cellulose and xyloglucan^26^. Moreover, EDX analysis confirmed that the nanocellulose is free from alkali or other residual elements and thereby also confirmed the purity of the sample to a reasonable extent. As shown in Table 3 oxygen was detected as the main element in nanocellulose besides carbon. Sulfur and silica were also detected but the value was insignificant, and also Aluminum and Iron are present in very trace amounts. Sulfur is left over from the preparation of nanocellulose using sulfuric acid, as previously described, and silica and other elements are left over from *Ulva lactuca*, as it was found in previous studies^57^.

**Table 3:** Chemical compositions obtained from EDX for nanocellulose.

Elements	Weight%	Atomic%
C K	42.66	49.06
O k	50.64	46.67
S K	4.60	3.08
Si K	1.26	0.71
Al K	0.8	0.47
Fe K	0.04	0.01

#### X-ray diffraction for cellulose and nanocellulose

**Figure 5:**
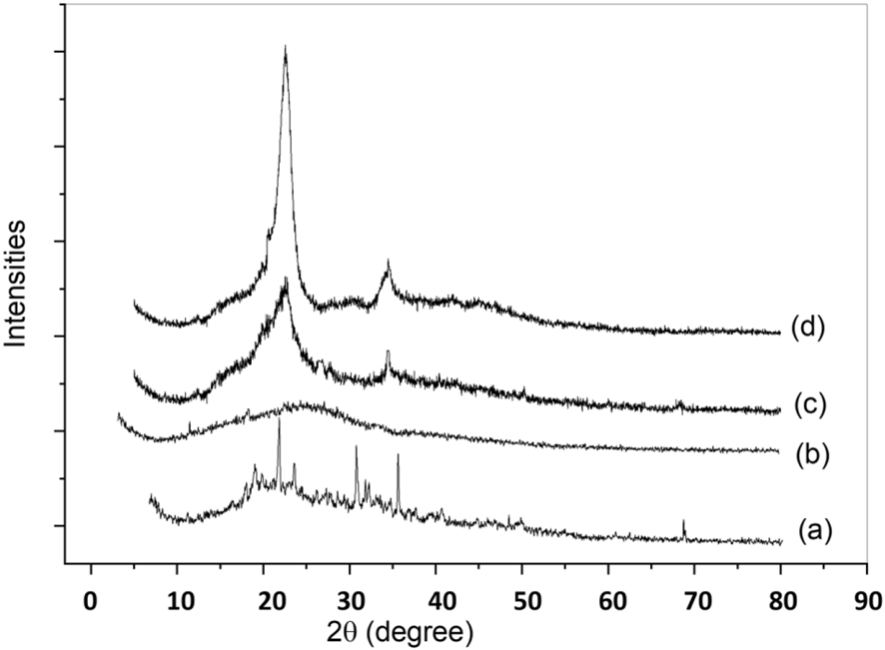
XRD (a) *Ulva lactuca*, (b) nanocellulose, (c) extracted cellulose, (d) reference cellulose.

Figure 5 demonstrated XRD bands, which publicized the crystallinity quality of reference cellulose, isolated cellulose from *Ulva lactuca,* and nanocellulose, respectively in addition to the XRD chart of *Ulva lactuca*. The extracted cellulose and nanocellulose both showed a small shift in the position of their major peaks, with the latter moving up by about 2 theta. Figure 5a shows that after chemical pretreatment, the crystallinity of the *Ulva lactuca* increased, as indicated by a narrower peak width and a greater number of identified cellulose crystal planes compared to those of untreated *Ulva lactuca*. This may be evidence that amorphous constituents present in untreated *Ulva lactuca* were partially removed^58^. Strident peaks from 0° to 60° at 2θ were pragmatic in the XRD design, Diffraction peaks at 2θ equal 15.9°, 22.6°, 23.1, and 32.6° were observed for all three samples; these angles correspond to the (100), (110), (110) and (111) planes of cellulose design^59^. It was concluded that the crystalline cellulose phase may be successfully extracted via chemical treatments, implying that lignin, hemicellulose, and other non-cellulosic components are removed efficiently from their amorphous phase of existence^38^.

This finding further demonstrates that the cellulose in Ulva lactuca residue retained its crystalline nature despite being referred to as acid hydrolysis. After amorphous sections of Ulva lactuca structure were chemically cleaved, no discernible deflection peaks of amino acid, hemicellulose, lignin, amorphous and defective cellulose crystals, and in addition to other parts. In comparison to isolated cellulose fibers, nanocellulose is far more potent. It is the amorphous parts that acid hydrolysis affects. Due to the large spread around the value of Iam, we were unable to determine the crystallinity index for the revived cellulose fraction.

The XRD pattern was used to determine the crystallinity index (Crl %) with the help of the Segal equation. Since the value of I_am_ could not be deduced from the broad peak at 21Ө, the crystallinity index for the extracted cellulose could not be determined. A sample with such a large peak is likely to be amorphous. This could be the reason why the TGA study showed that the thermal stability of the *Ulva lactuca* was lower than that of the extracted cellulose. According to Table 4, the hydrolysis process did not significantly alter the crystallinity of the extracted cellulose and nanocellulose, which had a crystallinity index of 58%. *Ulva lactuca*'s amorphous sections of cellulose were partially eliminated by an acidic treatment, leading to an increase in the crystallinity index after further treatment stages. Due to the presence of xyloglucan in the samples, it is clear why both the extracted cellulose and the nanocellulose have a mild degree of crystallinity. In addition, the particles of nanocellulose have a size of less than 50 nm as shown in Table 5.

**Table 4:** The crystallinity index (Crl) of reference cellulose, extracted cellulose, nanocellulose and *Ulva lactuca.*

Sample	I_200_	I_am_	Crl(%)
Reference cellulose	1745	570	67.33
Extracted cellulose	1240	520	58.06
Nanocellulose	1240	520	58.06
*Ulva lactuca*	670	450	32.83

**Table 5:** X-ray diffraction analysis, simple peak index, and size of nanocellulose.

2θ of the intense peak	d	1000/d^2^	(1000/d^2^)/32.5	hkl	FWHM (β) radians	Size of the particles (D) nm
15.954	5.55073	32.4563	1	100	0.0059	24.46
22.661	3.9208	65.0504	2	110	0.0066	22.77
23.118	3.84427	67.6664	2	110	0.0054	27.96
32.621	3.01338	110.1261	3	111	0.0048	33.25

hkl: Miller indices are proportional to the inverses of the intercepts of the plane, d:distance between the planes described by hkl.

### Investigation of the experiment organisms

**Figure 6:**
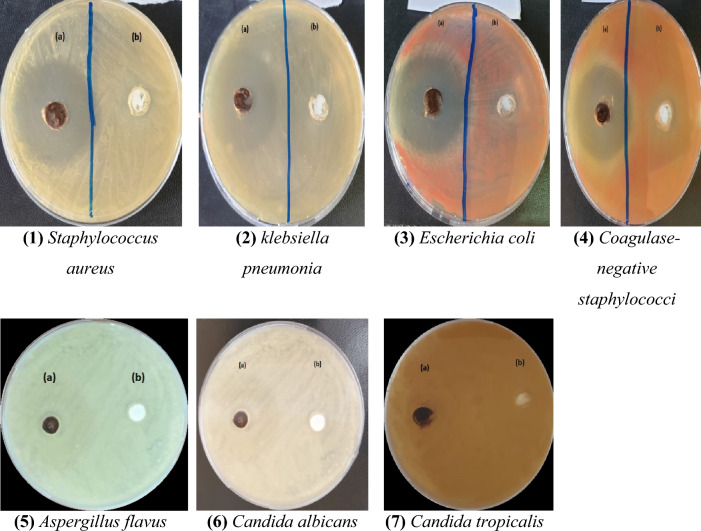
antimicrobial action of (a) nanocellulose (b) cellulose against test bacteria.

The antibacterial susceptibility test was performed on Mueller–Hinton agar for bacteria and Sabouraud Dextrose Agar (SDA) for fungi by using the well diffusion agar method against Gram (+ve) bacteria such as *Staphylococcus aureus* (ATCC6538) and *Klebsiella pneumonia* (ST627) and Gram (−ve) bacteria such as *Escherichia coli* (ATCC25922) and *coagulase-negative staphylococci (CoNS)* and some fungi such as *Aspergillus flavus, Candida albicans,* and *Candida tropicalis* as exposed in Fig. 6, where you can see the diameter of the area in each plate.

**Figure 7:**
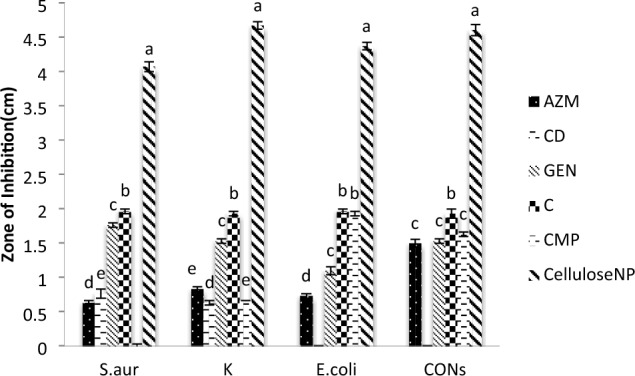
Compares between antibacterial action of nanocellulose and some antibiotics such as Azithromycin (AZM), Clindamycin (CD), Gentamicin (GEN), Chloramphenicol (C), and Cefepime (CMP) against *Staphylococcus aureus* (*S.aur*), *Klebsiella pneumonia* (k), *Escherichia coli* (*E.coli*), and Coagulase-negative staphylococci (CONs). a, b, c, d, e Statistical significant difference (P < 0.05), a > b > c > d > e.

**Figure 8:**
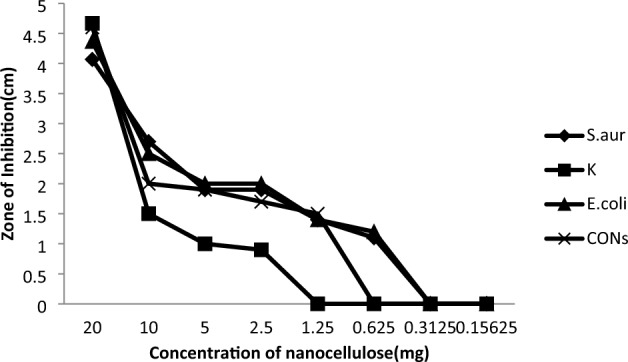
Minimum inhibitory concentration value of nanocellulose against *Klebsiella pneumonia* (k), *Staphylococcus aureus* (*S.aur*), *Escherichia coli* (*E.coli*), and Coagulase-negative staphylococci (CONs).

Since both Gram-positive (G+) and Gram-negative (G−) bacterial cell walls retain a negative charge due to the presence of phosphate groups in the peptidoglycan and phospholipids of their outer membranes, nanocellulose materials have an inherent antibacterial ability^60^. Despite the amazing effect of nanocellulose on bacteria, it did not show any effect on the types of fungi used in this research, so we made a comparison between the result of nanocellulose and some different antibiotics on bacteria which are mentioned in Fig. 7, we found that the effect of nanocellulose is stronger than any tested antibiotic where the antibacterial effect was significantly decreased in comparison with antibiotics used in our research. The evaluation of minimum inhibitory concentrations (MIC) of NC against *Staphylococcus aureus* (ATCC6538), *Klebsiella pneumonia* (ST627), *Escherichia coli* (ATCC25922), and *coagulase-negative staphylococci (CoNS)* were 0.31, 1.25, 0.31 and 0.625 mg/mL respectively were occurred by disk diffusion method as shown in Fig. 8.

## Conclusion

In this study, green macroalga *Ulva lactuca* was used to successfully isolate cellulose obtained from the Abo Qir beach in Alexandria, Egypt, by serial treatment, the cellulose content of the *Ulva lactuca* was 90.2%, then prepared nanocellulose from extracted cellulose which gives the same peaks in FTIR and XRD analysis in comparison with reference cellulose and nanocellulose was manufactured in conformity with the specifications of nanocellulose recognized in scientific research in a small size that does not exceed 50 nm, especially after sonication. The crystallinity index of the obtained nanocellulose was 68.7%. Characterization of the morphology of extracted cellulose and nanocellulose by SEM shows a combination of nanofibrils and regions with a slab-like shape. Nanocellulose is stable in temperatures up to 320 °C and contains low inorganic ash, as demonstrated by thermal studies. Furthermore, the obtained nanocellulose showed good thermal stability. The CNC obtained from the biomass, *Ulva lactuca*, showed great potential as antimicrobial which achieves tremendous challenges against Gram+ bacterium like *Staphylococcus aureus* (*ATCC6538*), *Klebsiella pneumonia* (*ST627*)*,* and Gram-negative bacterium such as *Escherichia coli (ATCC25922)* and *coagulase-negative staphylococci (CoNS)* especially when observed the result when tested this species with some antibiotics such as Azithromycin (AZM), Clindamycin (CD), Gentamicin(GEN), Chloramphenicol (C) and Cefepime (CMP) we found that the effect of nanocellulose on tested bacteria is higher than the effect of these antibiotics. According to this data, we can use nanocellulose as the main material in wound dressing, medical staff clothing, and protective antimicrobial suits because of the release of antimicrobial agents. The antibacterial activity of nanocellulose is also dependent on intrinsic and extrinsic factors. Intrinsic factors include the molecular weight and degree of extraction of parent cellulose, size, and concentration of nanocellulose. The extrinsic factors involve the sort of bacteria, temperature, and handling accuracy. So we can say nanocellulose is a sustainable, environmentally friendly material.

## Data Availability

All data and materials are included within the manuscript.
